# Supplementary material for: Do positive and negative shocks of tourism development affect income inequality in a developed country?
Source: PLoS One. 2024 Apr 16;19(4):e0301628. doi: 10.1371/journal.pone.0301628 (PMC11020903; doi:10.1371/journal.pone.0301628)
Supplement: Attachment — Submitted filename: 7 march report PONE-D-24-03256.pdf [file pone.0301628.s001.pdf]

PLOS ONE

Do positive and negative shocks of tourism development affect income inequality in a developed country?

--Manuscript Draft--

**Manuscript Number:**	PONE-D-24-03256
**Article Type:**	Research Article
**Full Title:**	Do positive and negative shocks of tourism development affect income inequality in a developed country?
**Short Title:**	Tourism development affect income inequality in Singapore
**Corresponding Author:**	Ngoc Bui Hoang, Ph.D<br>Ho Chi Minh City Open University<br>Ho Chi Minh, Tan Phu District VIET NAM
**Keywords:**	Tourism Development; Income Inequality; human capital; Asymmetric effect; Singapore.
**Abstract:**	Income inequality is an essential cause of violence, stagnant development, and political instability. Many economic and social policies were suggested, however, eliminating inequality seems to be a key challenge for policy-makers in both developed and developing countries. By adopting autoregressive distributed lag and non-linear autoregressive distributed lag approaches, this study aims to identify the potential asymmetric effect of tourism development on income inequality (measured by the Gini coefficient) in Singapore from 1978 to 2019. The results indicate that both positive and negative changes in tourism development lead to decreased income inequality. More specifically, the asymmetric effect of tourism is found both in the short- and long-term, and the positive changes have a greater impact than the negative changes. Moreover, economic growth, globalization, and human capital are drivers of income inequality in Singapore. These findings strengthen the belief of Singapore policy-makers and recommend several significant lessons for developing countries to promote tourism, sustainable development, and reduce income inequality.
**Order of Authors:**	Ngoc Bui Hoang, Ph.D
**Additional Information:**	
**Question**	**Response**
**Financial Disclosure**<br><br>Enter a financial disclosure statement that describes the sources of funding for the work included in this submission. Review the submission guidelines for detailed requirements. View published research articles from *PLOS ONE* for specific examples.<br><br>This statement is required for submission and **will appear in the published article** if the submission is accepted. Please make sure it is accurate.	The author(s) received no specific funding for this work.

Powered by Editorial Manager® and ProduXion Manager® from Aries Systems Corporation

**Funded studies**<br>Enter a statement with the following details:<br>• Initials of the authors who received each award<br>• Grant numbers awarded to each author<br>• The full name of each funder<br>• URL of each funder website<br>• Did the sponsors or funders play any role in the study design, data collection and analysis, decision to publish, or preparation of the manuscript?<br><br>Did you receive funding for this work?	
**Competing Interests**<br><br>Use the instructions below to enter a competing interest statement for this submission. On behalf of all authors, disclose any competing interests that could be perceived to bias this work—acknowledging all financial support and any other relevant financial or non-financial competing interests.<br><br>This statement is required for submission and **will appear in the published article** if the submission is accepted. Please make sure it is accurate and that any funding sources listed in your Funding Information later in the submission form are also declared in your Financial Disclosure statement.<br><br>View published research articles from *PLOS ONE* for specific examples.	The authors have declared that no competing interests exist.

**NO authors have competing interests**<br><br>Enter: *The authors have declared that no competing interests exist.*<br><br>**Authors with competing interests**<br><br>Enter competing interest details beginning with this statement:<br><br>*I have read the journal's policy and the authors of this manuscript have the following competing interests: [insert competing interests here]*<br><br>* typeset	
**Ethics Statement**<br><br>Enter an ethics statement for this submission. This statement is required if the study involved:<br><br>• Human participants<br>• Human specimens or tissue<br>• Vertebrate animals or cephalopods<br>• Vertebrate embryos or tissues<br>• Field research<br><br>Write "N/A" if the submission does not require an ethics statement.<br><br>General guidance is provided below. Consult the submission guidelines for detailed instructions. **Make sure that all information entered here is included in the Methods section of the manuscript.**	N/A

Powered by Editorial Manager® and ProduXion Manager® from Aries Systems Corporation

**Format for specific study types**<br><br>**Human Subject Research (involving human participants and/or tissue)**<br>• Give the name of the institutional review board or ethics committee that approved the study<br>• Include the approval number and/or a statement indicating approval of this research<br>• Indicate the form of consent obtained (written/oral) or the reason that consent was not obtained (e.g. the data were analyzed anonymously)<br><br>**Animal Research (involving vertebrate animals, embryos or tissues)**<br>• Provide the name of the Institutional Animal Care and Use Committee (IACUC) or other relevant ethics board that reviewed the study protocol, and indicate whether they approved this research or granted a formal waiver of ethical approval<br>• Include an approval number if one was obtained<br>• If the study involved *non-human primates*, add *additional details* about animal welfare and steps taken to ameliorate suffering<br>• If anesthesia, euthanasia, or any kind of animal sacrifice is part of the study, include briefly which substances and/or methods were applied<br><br>**Field Research**<br><br>Include the following details if this study involves the collection of plant, animal, or other materials from a natural setting:<br>• Field permit number<br>• Name of the institution or relevant body that granted permission	
**Data Availability**<br><br>Authors are required to make all data underlying the findings described fully available, without restriction, and from the time of publication. PLOS allows rare exceptions to address legal and ethical concerns. See the PLOS Data Policy and FAQ for detailed information.	Yes - all data are fully available without restriction

A Data Availability Statement describing where the data can be found is required at submission. Your answers to this question constitute the Data Availability Statement and **will be published in the article**, if accepted.<br><br>**Important:** Stating 'data available on request from the author' is not sufficient. If your data are only available upon request, select 'No' for the first question and explain your exceptional situation in the text box.<br><br>Do the authors confirm that all data underlying the findings described in their manuscript are fully available without restriction?	
**Describe where the data may be found in full sentences. If you are copying our sample text, replace any instances of XXX with the appropriate details.**<br><br>• If the data are **held or will be held in a public repository**, include URLs, accession numbers or DOIs. If this information will only be available after acceptance, indicate this by ticking the box below. For example: *All XXX files are available from the XXX database (accession number(s) XXX, XXX.*).<br>• If the data are all contained **within the manuscript and/or Supporting Information files**, enter the following: *All relevant data are within the manuscript and its Supporting Information files.*<br>• If neither of these applies but you are able to provide **details of access elsewhere**, with or without limitations, please do so. For example:<br><br>*Data cannot be shared publicly because of [XXX]. Data are available from the XXX Institutional Data Access / Ethics Committee (contact via XXX) for researchers who meet the criteria for access to confidential data.*<br><br>*The data underlying the results presented in the study are available from (include the name of the third party*	We confirm that all sources of data were presented in the paper. We do not own data, and we do not have any special access privileges that others would not have.

and contact information or URL).<br>• This text is appropriate if the data are owned by a third party and authors do not have permission to share the data.<br><br>* typeset	
Additional data availability information:	

Manuscript Click here to access/download;Manuscript;Full manuscript.docx

DO POSITIVE AND NEGATIVE SHOCKS OF TOURISM DEVELOPMENT AFFECT INCOME INEQUALITY IN A DEVELOPED COUNTRY?

Bui Hoang Ngoc[1]

1. Finance, Economic and Management Research Group, Ho Chi Minh City Open University, Postal address: 97 Vo Van Tan Street, District 3, Ho Chi Minh City 700000, Vietnam.
   Corresponding email: ngoc.bh@ou.edu.vn
   Link ORCID: https://orcid.org/0000-0002-1892-985X

Acknowledgment: We thank the anonymous referees and Editor-in-chief for their careful reading of our manuscript and their many insightful comments and suggestions that improved the quality of the initial manuscript. Any remaining errors are our sole responsibility.

Fundings: No funding was received by the author for this research.

Disclosure statement: No potential conflict of interest was reported by the authors.

Data source

We confirm that all sources of data were presented in the paper. We do not own data or have any special access privileges that others would not have. The data of this study can be downloaded from: https://www.singstat.gov.sg/

Abstract

Income inequality is an essential cause of violence, stagnant development, and political instability. Many economic and social policies were suggested, however, eliminating inequality seems to be a key challenge for policy-makers in both developed and developing countries. By adopting autoregressive distributed lag and non-linear autoregressive distributed lag approaches, this study aims to identify the potential asymmetric effect of tourism development on income inequality (measured by the Gini coefficient) in Singapore from 1978 to 2019. The results indicate that both positive and negative changes in tourism development lead to decreased income inequality. More specifically, the asymmetric effect of tourism is found both in the short- and long-term, and the positive changes have a greater impact than the negative changes. Moreover, economic growth, globalization, and human capital are drivers of income inequality in Singapore. These findings strengthen the belief of Singapore policy-makers and recommend several significant lessons for developing countries to promote tourism, sustainable development, and reduce income inequality.

Keywords: Tourism development; Income inequality; Human capital; Asymmetric effect; Singapore.

JEL: F43, O13, O47, Q42, Q43.

1. Introduction

Income inequality (IE) is a social problem, and it is considered an undesirable consequence of economic policies issued by the government over a long time. Unfortunately, it has increased at varied rates in almost all countries [1]. Income inequality develops depending on the government's perspective, level of awareness, and residents' opposition. The widening gap between the rich and poor might limit the opportunities of poorer people and, over time, might cause violence and social and political instability [2, 3]. Therefore, reducing income inequality and then wholly eliminating it is the desire of many governments and people worldwide. Thus, economic policies that help reduce income inequality are always sought and welcomed [4-6]. Tourist development (TO) is often expected to be an effective policy for alleviating poverty because it brings a multiplicity of benefits to a country, such as promoting infrastructure, attracting foreign direct investment, generating new jobs, and stimulating cultural change, thereby it could help reduce income inequality [4, 5, 7, 8]. However, Qiao and Chen [9] and Carrascal Incera and Fernández [10] revealed that a significant part of the labor force in tourism is related to self-employment or family enterprises. Njoya and Seetaram [11] also noted that TO requires investment and management experience, and poor people do not have these inputs. In some cases, a significant portion of tourism revenue may leak out of the local economy through imports, foreign-owned businesses, or multinational hotel chains. This can limit the trickle-down effect of tourism revenue and exacerbate income inequality [4]. Notably, Kinyondo and Pelizzo [12] argued that tourism has long been seen as a source of social inequality in Kenya, while Khan, Bibiet al. [13] revealed that there is a relationship between TO, terrorism, and inequality in Pakistan. Therefore, a comprehensive understanding of the impact of tourism on IE and its consequences has profound implications for policy-makers in planning strategies that prioritize local participation, equitable distribution of tourism benefits, and protect the rights and livelihoods of the poor.

Unfortunately, the debate on whether tourism can help reduce inequality is still going on. Studies on this topic have different conclusions and are not definitive. For example, Uzar and Eyuboglu [14], Porto and Espinola [15] found that tourism development increases inequality, while Nguyen, Schinckuset al. [3], Fang, Gozgoret al. [6], Shahbaz, Solarinet al. [16] suggested that tourism can lead to fairer income distribution. Another study by Fang, Gozgoret al. [6] revealed that TO significantly negatively influences IE in developing countries and has an insignificant impact in developed nations. Consequently, some researchers are studying how

tourism development affects income distribution by looking at different factors like income, tax, price, and risk. The goal of this exploration is to fully comprehend how tourism impacts economic and social aspects of a country. Some reasons are given to explain the impact of TO on IE, such as (*i*) tourism can reduce income inequality by creating jobs for the poorest workers. However, tourism development may also lead to higher income inequality by increasing demand for infrastructure, benefiting the wealthier population [6]; (*ii*) the relationship between tourism and income distribution depends on institutions. A better institutional environment and a suitable tax policy can boost tourism opportunities, and in turn, tourism activities can also contribute to creating a more favorable institutional environment [3].

Singapore presents itself as a captivating context for investigating this association. In 2020, the tourism industry generated around 527,500 jobs and contributed SGD 52.5 billion to the economy [17]. Singapore's tourism development is an enticing example for emerging countries, as it enhances per capita income. However, examining whether tourism development in Singapore also impacts income inequality is crucial to fully comprehend tourism's influence on macroeconomic variables. Singapore, currently ranked among the top 10 countries in the world with the highest income, boasts an impressive per capita income of $60,729 per year (at the fixed price of 2010). However, it is important to note that such prosperity does not necessarily address the prevailing income disparity between the affluent and the less fortunate within Singapore. Startling figures from a recent report on income inequality indicate that in 2010, the income gap between the higher and lower-income classes was $9,288. Shockingly, this gap has widened to $12,840 in 2020, highlighting a growing challenge of eradicating inequality for Singaporean administrators.

Notably, the interconnections between tourism development and income inequality were often analyzed by a linear framework. For example, Oviedo-García, González-Rodríguez and Vega-Vázquez [18] applied the autoregressive distributed lag (ARDL) approach to inspect the interaction between TO and IE, and indicated that TO reduce IE in the Dominican Republic. A similar conclusion can be found in a study by Subramaniam, Masron and Loganathan [4]. However, tourism development can be influenced by various factors, such as pandemics [19-21], institutions [3], and financial development [22]. So, maintaining the continuous growth of the tourism sector is unrealistic, which implies that the linear framework may provide an incomplete picture and can not fully explain the relationship between TO and IE. Furthermore, Khoi, Le and Ngoc [23] recently found that the impact of TO on ecological footprint in Singapore is

asymmetric. Katrakilidis and Trachanas [24], and Ahmed, Zhang and Cary [25] pointed out that if the relationship between these variables is asymmetric, the policy implications based on the linear framework could be unreliable. Hence, this work aims to explore the role of tourism in reducing IE in Singapore by considering economic growth, globalization, and human capital in which tourism activities occur. The motivation and contribution of this study are summarized in detail:

(i) Despite the abundance of previous studies on links between tourism and income inequality, the negative and positive changes in tourism development that might lead to effects of different magnitude or directions in income have been ignored. If the nexus between TO and IE is asymmetric, the policy implications based on symmetric methods can be unreliable. This study employs the linear ARDL and non-linear ARDL approaches to identify a symmetric or asymmetric effect of tourism on income inequality to provide a comprehensive understanding of tourism development in Singapore.

(ii) Globalization and human capital are considered the keys to tourism development. However, the impact of two factors on income inequality is still fragmented and unclear in Singapore. In this study, globalization and human capital play as control variables in identifying the tourism-income inequality nexus.

(iii) From 1978 to 2019, Singapore's economy suffered two financial crises (in 1997 and 2008), implying that the data might have some structural breaks. Therefore, an advanced unit root technique was also employed to probe structural breaks in the data, which is superior to many other tests regarding size and accuracy in identifying breaks. Along with the results obtained from the non-linear ARDL approach, the findings of this study strengthen administrators' belief in promoting tourism and reducing income inequality in Singapore.

2. Literature review

Economic growth and income inequality

A fundamental question in sustainable development policies is whether economic growth retards or accelerates income inequality. The pioneering study of Kuznets [26] predicted that there is an inverted U-shape relationship between economic growth and income inequality. Following this prediction, many scholars investigated whether, how, and to what extent economic growth could affect income distribution. However, the answer remains elusive. A study that

found a negative relationship is the study of Braun, Parro and Valenzuela [27], who conducted research that evaluated the major prediction of their model in terms of the influence of income disparity on growth at various degrees of financial development. They discovered that greater income inequality is related to weaker economic development by utilizing pooled OLS, FEM, and REM approaches in 150 countries between 1978 and 2012. Furthermore, they discovered that as economies' financial development levels rise, such an influence becomes substantially less pronounced.

In another study, Royuela, Veneri and Ramos [28] examined the relationship between income inequality and growth in 15 OECD nations from 2003 to 2013. It integrates household survey data and macroeconomic statistics from 15 OECD nations to cover over 200 similar areas. Their econometric findings showed an overall negative relationship between disparities and economic development since the beginning of the economic crisis. The sort of urban structure influences this connection. Higher disparities appear to be more destructive to growth in regions typified by medium to large-sized cities, whereas small cities and rural areas appear to be less affected. Likewise, Breunig and Majeed [29] recently re-examined the relationship between inequality and economic development in 152 countries. The study employed GMM and discovered that inequality had a detrimental influence on growth from 1956 to 2011. They also discovered that when both poverty and inequality were taken into account, the negative impact of inequality on growth was concentrated in nations with high poverty rates. More precisely, they found that when poverty (daily income below US$ 1.9), the impact of inequality on economic growth is greater.

Some studies show a positive relationship between economic growth and income inequality. Scholl and Klasen [30] reviewed the inequality-growth link, focusing on the significance of transition (post-Soviet) nations. They discovered a positive relationship between inequality and growth in the total sample using FE, GMM, and IV estimation methodologies driven by transition nations. Brueckner and Lederman [31] researched the connection between inequality and GDP per capita growth. Using panel data from 1970 to 2010, the researchers discovered that increased income disparity boosted transitory growth in low-income countries while having the opposite impact in high-income nations. However, Benos and Karagiannis [32] investigated the link between top income inequality and growth in the United States due to physical and human capital buildup. They determined that increases in inequality did not affect growth by utilizing 2SLS and GMM on an annual panel of US state-level data from 1929 to 2013.

Tourism development, globalization, human capital and income inequality

Some studies showed that tourism contributes to increasing income inequality. In particular, Chi [33] indicated that the long-run link between tourist revenue and income inequality differs across industrialized and developing nations. In developing countries, income disparity worsens as tourist revenue grows, improves after the first turning point is achieved, and worsens again after the second turning point. Similarly, Oviedo-García, González-Rodríguez and Vega-Vázquez [18] revealed that tourist income has not relieved poverty and has obviously failed to lessen wealth disparity.

It is plausible that activities associated with tourism might have varying effects on economic disparity, to the point that it has been statistically demonstrated that domestic tourism can make income disparity worse, while foreign tourism can reverse the situation (Nguyen et al., 2021). In contrast to the common idea that tourism contributes to economic expansion and reduces poverty levels, Zhang's study suggests that more tourism leads to greater income disparity (Zhang, 2021), especially in developing countries (Alam & Paramati, 2016). However, Uzar & Eyuboglu (2019) and Li et al., (2016) assert that tourism helps alleviate economic inequality, and Turkey's tourism is substantiated to affect income distribution. In Raza and Shad's paper, with the proviso that the countries make the expansion of their tourism industries a priority, they will be able to reduce the economic inequality that exists in the region (Raza & Shah, 2017). Thus, it is necessary to identify whether Singaporean tourism has a symmetric or asymmetric influence on its income inequality.

One of the reported advantages of globalization is that it encourages the development of new employment, which, in turn, leads to an improvement in the standard of living of people all over the world and a reduction in poverty [34]. While Ghosh and Mitra [35] indicate that the distribution of income within highly developed countries is mostly unaffected by the growth of the tourism industry, [36] emphasize that more globalization causes a decline in the economic discrepancy that exists in that country. On the other hand, globalization magnifies the distribution of wealth by creating a skills gap in corporate operations, which in turn leads to a wider disparity in income [37]. By the same token, globalization has significantly widened the income gap between the rich and the poor in developing countries [6]. Therefore, globalization acts as a control variable in determining the relationship between tourism and income inequality.

According to social scientists, the term "human capital" refers to the attributes of a person that are deemed valuable to the production process. These attributes might include knowledge,

skills, expertise, good health, education, and so on. The findings of Tsaurai [38] reverberate the findings of Becker [39], who noted that high levels of human capital development mean that individuals are talented, educated, and trained. They are also believed to be more productive, more marketable, and more likely to find a job that pays a higher salary. Therefore, investments in human capital development will allow developing nations to nurture a labor force of better quality and capable of maximizing earnings from tourism [35].

Some scholars confirmed the adverse effects of human capital and income inequality. Some examples include Ajide and Alimi [40], who found that the marginal effect of interactions between human capital measures and income inequality indicators is negative. Hu [2] pointed out the influence of income disparity on human capital inequality in 31 Chinese provinces, municipalities, and autonomous areas from 1996 to 2018. On the contrary, Chani, Janet al. [41] confirmed the presence of a long-run link and the causal relationship between human capital inequality and income inequality, and the Johanson cointegration and Granger causality tests are utilized. Suhendra, Istikomahet al. [42] used a panel data model with a fixed effect estimate for data from 34 Indonesia's provinces from 2013 to 2019 and found that human capital has a negative and significant effect on income inequality. According to the above criteria, the authors manually screened the collected literature in Table 1.

Table 1. A summary of available studies on the relationship between tourism development, economic growth, globalization, human capital, and income inequality.

No.	Authors	Duration	Countries/Areas	Econometric approach	Findings
**Tourism development – income inequality**					
1	Ofori, Dossou and Akadiri [43]	1996 - 2020	48 African countries	GMM	
2	Ghosh and Mitra [35]	1995 - 2016	41 countries	FMOLS	Developing Countries: - Developed countries: +
3	Fang, Gozgoret al. [6]	1995 - 2014	102 countries	Fixed-effects, FMOLS	Developing countries: -
4	Chi Chi [33]	1995 - 2015	20 developed countries and 16 developing countries	FMOLS, DOLS	Developing countries: +

5	Nguyen, Schinckuset al. [3]	2002 - 2014	97 countries	PCSE	-
6	Lv [44]	1995 - 2012	113 countries	FMOLS	-
7	Shahbaz, Solarinet al. [16]	1991 - 2017	Malaysia	ARDL	-
8	Oviedo-García, González-Rodríguez and Vega-Vázquez [18]	2000 - 2013	Dominican Republic	ARDL	-
9	Porto and Espinola [15]	2004 - 2015	Argentina	FEM, REM	+
**Economic growth – income inequality**					
1	Breunig and Majeed [29]	1956 - 2011	152 countries	GMM	-
2	Braun, Parro and Valenzuela [27]	1978 - 2012	150 countries	Pooled OLS	-
3	Royuela, Veneri and Ramos [28]	2003 - 2013	15 OECD countries	Pooled OLS<br>REM	-
4	Scholl and Klasen [30]	1961 - 2012	122 countries	FEM<br>GMM	+
5	Brueckner and Lederman [31]	1970 - 2010	144 countries,	2SLS<br>GMM	Low income countries: +<br>High income countries: -
6	Benos and Karagiannis [32]	1929 - 2013	US state level data,	2SLS<br>GMM	0
**Human capital – income inequality**					
1	Ofori, Dossou and Akadiri [43]	1996 - 2020	48 African countries	GMM	+
2	Ajide and Alimi [40]	1980 - 2012	34 African countries	Poisson regression	+
3	Hu [2]	1996 - 2018	31 provinces, China	Fixed effect, system GMM	+

4	Suhendra, Istikomahet al. [42]	2013 - 2019	4 provinces Indonesia	FEM, REM	+
5	Lee and Lee [45]	1980 - 2015	East Asian	VECM	+
6	Chani, Janet al. [41]	1973 - 2009	Pakistan	VAR model, and Granger causality	+

Notes: (+): positive impact; (-) negative impact.

Of course, none of the above studies and Table 1 could present the literature on the association between tourism development, economic growth, globalization, human capital, and income distribution. However, these studies also reveal that some natural issues have not been identified. One of the questions is whether the influence of TO on IE is symmetric or asymmetric. In Singapore, Khoi, Le and Ngoc [23] also indicated that the impact of TO on ecological footprint is asymmetric. Chishti, Ullahet al. [46] also reported that positive changes in TO ameliorate the environmental quality by reducing carbon emissions in Nepal and Sri Lanka, and increasing the carbon emissions in Bangladesh, India, and Pakistan. In addition, the asymmetric effect of other macroeconomic variables, such as financial development [47], foreign direct investment [48], energy consumption [49, 50], and exchange rate [51], could be found in previous studies. That means linear models may not be appropriate to explain the impacts of TO, and raise doubt about the effectiveness of suggested policies. From a methodological standpoint, an analysis including linear and non-linear frameworks gives a comprehensive understanding of the role and marginal effect of tourism shock on income inequality. Therefore, this study enriches the literature and provides a complete picture of the relationship between TO and IE in Singapore.

3. Methodology

Several factors affect the links between tourism - income inequality nexus, such as institutions [3], urbanization [47], human capital [48], and globalization [36]. Based on these studies, to identify the asymmetric effects of TO on income inequality in Singapore, our model is proposed in detail:

$$IE_t = \alpha_0 + \alpha_1.\ln TO_t + \alpha_2.\ln GDP_t + \alpha_3.HC_t + \alpha_4.Glob_t + e_t \quad \text{(Eq.1)}$$

where, the *IE* variable is income inequality (measured by Gini coefficient), and TO is measured by the number of international visitors. *GDP*, *HC*, and *Glob* variables are economic growth, human capital, and globalization. Two variables (*TO* and *GDP*) are transformed into natural logarithms to smooth data, while *IE*, *HC*, and *Glob* are used as original data. Annual data

is given by the Department of Statistics Singapore (for tourism, economic growth variable), Swiss Institute of Economics (globalization), and Federal Reserve Economic Data (human capital) from 1978 to 2019.

To examine the long-run association among the study variables, following the ARDL approach introduced by Pesaran and Shin [49], Eq.1 can be written as:

$$\begin{aligned}\Delta IE_t &= \beta_0 + \beta_1.IE_{t-1} + \beta_2.\ln TO_{t-1} + \beta_3.\ln GDP_{t-1} + \beta_4.HC_{t-1} + \beta_5.Glob_{t-1} + \\ &+ \sum_{j=1}^{p-1}\alpha_{1j}\Delta IE_{t-j} + \sum_{j=0}^{q}\alpha_{2j}\Delta \ln TO_{t-j} + \sum_{j=0}^{q}\alpha_{3j}\Delta \ln GDP_{t-j} + \sum_{j=0}^{q}\alpha_{4j}\Delta HC_{t-j} + \\ &+ \sum_{j=0}^{q}\alpha_{5j}\Delta Glob_{t-j} + \varepsilon_t\end{aligned} \qquad \text{(Eq.2)}$$

where, Δ represents the first difference, and ε is the error term. β_i (i = 1,..,5) are long-run coefficients, while α_i (i = 1,..,5) are short-run coefficients.

To probe asymmetric effects, the study follows the suggestion of Shin, Yu and Greenwood-Nimmo [50] to separate negative and positive changes in TO variable, as specific:

$$\ln TO_t = \ln TO_0 + \ln TO_t^+ + \ln TO_t^-$$

where, $\ln TO_0$ is the minimum number of international visitors, $\ln TO_t^+$ and $\ln TO_t^-$ are partial sum processes of the positive and negative changes in tourism, which are calculated as details:

$$\ln TO_t^+ = \sum_{j=1}^{t}\Delta \ln TO_j^+ = \sum_{j=1}^{t}\max(\Delta \ln TO_j, 0)$$

$$\ln TO_t^- = \sum_{j=1}^{t}\Delta \ln TO_j^- = \sum_{j=1}^{t}\min(\Delta \ln TO_j, 0)$$

Therefore, Eq.2 can be written as a non-linear ARDL(p,q) approach, as follows:

$$\begin{aligned}\Delta IE_t &= \beta_0 + \beta_1.IE_{t-1} + \beta_2^+.\ln TO_{t-1} + \beta_2^-.\ln TO_{t-1} + \beta_3.\ln GDP_{t-1} + \beta_4.HC_{t-1} + \beta_5.Glob_{t-1} + \\ &+ \sum_{j=1}^{p-1}\alpha_{1j}\Delta IE_{t-j} + \sum_{j=0}^{q}\alpha_{2j}^+\Delta \ln TO_{t-j} + \sum_{j=0}^{q}\alpha_{2j}^-\Delta \ln TO_{t-j} + \sum_{j=0}^{q}\alpha_{3j}\Delta \ln GDP_{t-j} + \\ &+ \sum_{j=0}^{q}\alpha_{4j}\Delta HC_{t-j} + \sum_{j=0}^{q}\alpha_{5j}\Delta Glob_{t-j} + \varepsilon_t\end{aligned}$$

(Eq.3)

The null long-run asymmetric hypothesis is tested by H0,LR: $-(\beta_2^+/\beta_1) = -(\beta_2^-/\beta_1)$ against the alternative hypothesis H1,LR: $-(\beta_2^+/\beta_1) \neq -(\beta_2^-/\beta_1)$. Similarly, the null short-run asymmetric hypothesis is checked:

$$H_{0,SR} : \sum_{j=0}^{q} \alpha_{2j}^+ = \sum_{j=0}^{q} \alpha_{2j}^- \text{ against the alternative hypothesis } H_{1,SR} : \sum_{j=0}^{q} \alpha_{2j}^+ \neq \sum_{j=0}^{q} \alpha_{2j}^-$$

The asymmetric cumulative dynamic multiplier effects of a unit change in tourism on income inequality are specified:

$$m_h^+ = \sum_{j=0}^{h} \frac{\partial IE_{t+j}}{\partial \ln TO_t^+} ; m_h^- = \sum_{j=0}^{h} \frac{\partial IE_{t+j}}{\partial \ln TO_t^-} \text{ and h = 0,1,2….}$$

Note that as $h \to \infty$ then $m_h^+ \to \lambda_1^+, m_h^- \to \lambda_1^-$, where λ_1^+ and λ_1^- are the asymmetric long-run coefficients calculated as $\lambda_1^+ = -(\beta_2^+/\beta_1)$ and $\lambda_1^- = -(\beta_2^-/\beta_1)$, respectively.

4. Empirical results

Based on the advantages of geographic location and many effective tourism development policies, Singapore is one of Asia's leading international passengers nowadays. In 2019, Singapore experienced a record number of visitors (attracting 19.1 million international visitors), following strong year-on-year growth in visitor numbers since 2015 [17]. Even so, Singapore has managed to market itself as a must-visit tourist destination in its own right, attracting repeat visitors by offering unique city experiences such as the Gardens by the Bay, and integrated resorts boasting casinos and amusement parks. The descriptive statistics of variables are shown in Table 2.

Table 2. Descriptive statistic

Variables	Obs	Mean	Std.Error	Minimum	Maximum
IE	42	38.10	0.847	36.60	39.30
lnTO	42	15.74	0.631	14.53	16.76
lnGDP	42	10.28	0.488	9.34	10.98
HC	42	2.61	0.718	1.65	4.35
Glob	42	75.50	7.552	62.78	84.69

Table 2 shows that the highest value of income inequality is 39.30 percent, the human capital is 4.35 points, and the globalization index is 84.69 percent, but the lowest values in these three variables are 36.60, 1.65, and 62.78, respectively.

Unit root tests

According to Pesaran and Shin [49], it is necessary to check unit root before applying the ARDL approach because that any series are stationarity at the second diference I(2), the estimated coefficients will not be valid. Moreover, another issue of time series data anlysis is structural break. To accomplish this, the augmented Dicky-Fuller, and Lee & Strazicich tests [51] were employed. Results in Table 3 show that all variables are stationarity at the first difference, and no variables are stationarity at the second difference, which implies the first condition to apply ARDL and non-linear ARDL approach is satisfied [52]. Furthermore, the Lee & Strazicich test provides break years. However, the results are inconsistent, and it could not help choosing a fit break year. Table 3 also indicates that the optimal lag length of *IE* variable is three, while the optimal order of *lnGDP* and *HC* variables is two. Thus, the optimal ARDL model is ARDL(3,0,2,2,0).

Table 3. Results of the unit root test and structural breaks test

Variables	ADF test		Lee & Strazicich test	Break year	Optimal lag
	I(0)	I(1)			
IE	-2.097	-3.153**	-7.894***	1989 & 2004	3
lnTO	-2.538	-6.366***	-5.302	1995 & 2003	0
lnGDP	-2.050	-6.642***	-6.747***	1996 & 2008	2
HC	-0.147	-3.194*	-6.405***	1994 & 2007	2
Glob	-1.183	-5.649***	-6.681**	1999 & 2004	0

*Note: The ADF test is chosen based on the Akaike Information Criterion with intercept and trend. ***, ** and * respectively denote significance levels of 1%; 5% and 10%.*

Cointegration test

Table 4. Results of the cointegration test

F-Bounds test		Null hypothesis: No level relationship		
Test Statistic	Value	Signif	I(0)	I(1)
F-statistic in Eq.2	11.01	10%	2.68	3.53
k	4	5%	3.05	3.97
F-statistic in Eq.3	12.89	2.5%	3.40	4.36
k	4	1%	3.81	4.91

The findings of the unit root test indicated that our data series is integrated. Thus, the linear and non-linear bound test of cointegration was applied. The result of two tests in Table 4 reveals

that the F-statistic is greater than the upper critical bound at the level of 1 percent significance in Eq.2 and Eq.3. These outcomes allow the study to apply the ARDL and non-linear ARDL approaches in further analysis.

The short-run and long-run symmetric and asymmetric ARDL findings

The short-run and long-run linear ARDL findings are presented in Table 5, while the outcomes of non-linear ARDL are shown in Table 6. The coefficient of *lnTO* obtained from the symmetric ARDL approach is insignificant, implying no relationship between tourism and income inequality in Singapore. However, the results obtained from the asymmetric ARDL approach indicate that the influence of tourism on income inequality is evident. More specifically, the estimated coefficients of positive changes ($lnTO^+$) and negative changes ($lnTO^-$) are -0.896 and 0.471, respectively. The long-run asymmetric Wald test in the lowest of Table 6 provides evidence to reject the null hypothesis (W_{LR} = 53.79, *p-value* = 0.000).

Table 5. The symmetric ARDL findings

Dependent variable: ΔIE			
Variables	Coefficient	Standard error	T-ratio [Prob]
lnTO	-0.355	0.219	-1.62 [0.118]
lnGDP	4.762	0.545	8.73 [0.000]
HC	-0.960	0.181	-5.29 [0.000]
Glob	0.093	0.013	7.17 [0.000]
CointEq(-1)	-0.459	0.052	-2.49 [0.000]
$\Delta IE(-1)$	0.061	0.095	0.64 [0.527]
$\Delta IE(-2)$	0.236	0.087	2.72 [0.012]
$\Delta lnGDP$	1.089	0.198	5.50 [0.000]
$\Delta lnGDP(-1)$	-0.608	0.210	-2.89 [0.007]
ΔHC	-0.719	0.322	-2.23 [0.034]
$\Delta HC(-1)$	-0.833	0.333	-2.49 [0.019]
Intercept	-3.304	0.374	-8.83 [0.000]
R^2	0.9283	R^2-adj	0.9122
χ^2_{SC}	2.688 [0.088]	χ^2_{FF}	0.031 [0.862]
χ^2_{NORM}	0.872 [0.646]	χ^2_{HET}	1.128 [0.380]
CUSUM test	Stable	CUSUMSQ	Stable

Likewise, both estimated coefficients of $\Delta lnTO^{+}$ and $\Delta lnTO^{-}$ are significant, and the short-run asymmetric Wald test also rejects the null hypothesis (W_{SR} = 13.12, *p-value* = 0.001). These findings confirm that the impact of tourism development on income inequality is the asymmetry in both the short- and long-run. More specifically, a 1 percent increase in tourism leads to a 0.896 percent decrease in income inequality. Similarly, a 1 percent decrease in tourism leads to a 0.471 percent decrease in income inequality. Hence, the positive changes in tourism have a greater effect than negative changes. Figure 3 also confirms this conclusion.

In both symmetric and asymmetric ARDL approaches, the coefficients of lnGDP, HC, and Glob variables are significant and consistently influential. Specifically, economic growth and globalization positively impact income inequality, while an increase in human capital can potentially decrease income inequality. All diagnostic tests conducted in Tables 5 and 6, such as serial correlation, functional form, normality, and heteroskedasticity, have been successfully satisfied. Moreover, Figures 1 and 2 demonstrat[illegible]hat both the CUSUM and CUSUMSQ lines comfortably fall within the critical bounds at a significant level of 5 percent, implying that Eq.3 remains stable. With all the necessary conditions met in both symmetric and asymmetric ARDL approaches, this study ensures that the estimated results are not only reliable but also hold relevance for further analysis and the development of policy recommendations.

Table 6. The asymmetric ARDL findings

Dependent variable: ΔIE			
Variables	Coefficient	Standard error	T-ratio [Prob]
$lnTO^{+}$	-0.896	0.374	-2.39 [0.017]
$lnTO^{-}$	0.471	0.268	1.75 [0.079]
lnGDP	2.472	0.673	3.67 [0.000]
HC	-1.925	0.737	-2.61 [0.009]
Glob	0.087	0.018	4.73 [0.000]
CointEq(-1)	-0.432	0.045	-9.57 [0.000]
$\Delta IE(-1)$	-0.493	0.088	-5.58 [0.000]
$\Delta lnTO^{+}$	-0.442	0.183	-2.41 [0.023]
$\Delta lnTO^{-}$	0.232	0.127	1.83 [0.078]
$\Delta lnGDP$	1.221	0.329	3.82 [0.000]
$\Delta lnGDP(-1)$	0.939	0.359	2.61 [0.014]
ΔHC	-0.951	0.329	-2.88 [0.007]

ΔHC(-1)	0.129	0.359	0.36 [0.722]
ΔGlob	0.043	0.008	5.02 [0.000]
ΔGlob(-1)	0.003	0.011	0.27 [0.785]
Intercept	-3.786	1.931	-1.96 [0.059]
R^2	0.8903	R^2-adj	0.8525
χ^2_{SC}	3.702 [0.054]	χ^2_{FF}	0.001 [0.972]
χ^2_{NORM}	0.966 [0.270]	χ^2_{HET}	0.819 [0.532]
W_{LR}	53.79 [0.000]	W_{SR}	13.12 [0.001]

Note: The superscripts "+" and "-" denote positive and negative partial sums. χ^2_{SC}, χ^2_{FF}, χ^2_{NORM}, χ^2_{HET} denote LM tests for serial correlation, functional form, normality, and heteroskedasticity. The value in brackets is the corresponding p-value, respectively.

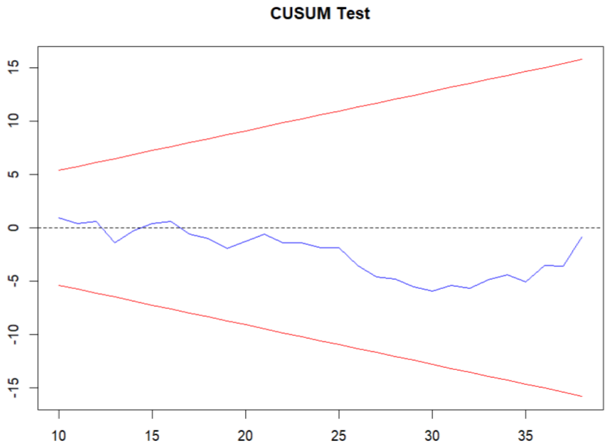

Figure 1. CUSUM test

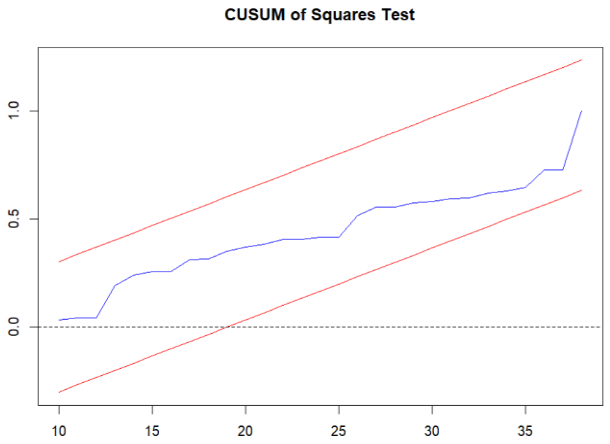

Figure 2. CUSUMSQ test

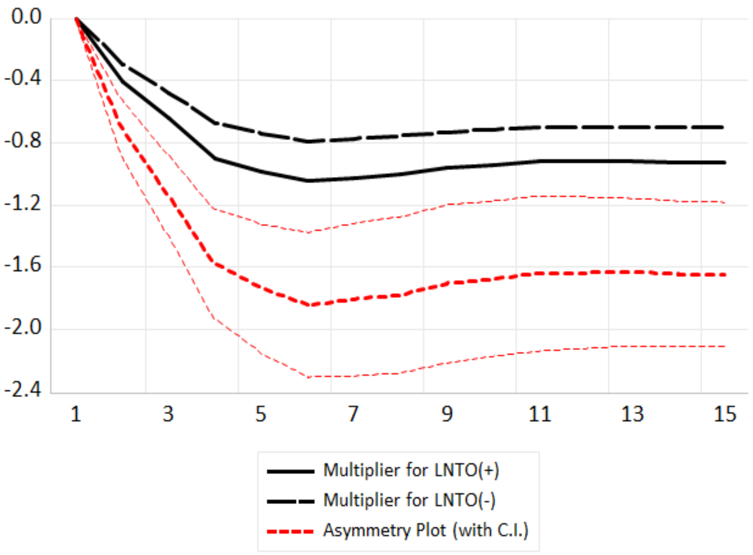

Figure 3. Dynamic multipliers for tourism development

Results of the causality test

The findings from ARDL and NARDL approaches have demonstrated the short- and long-run impacts of tourism development, economic growth, globalization, and human development on income inequality in Singapore. However, information about the causal relationship between these variables is also essential in suggesting lessons and policies. If the value of χ^2 (Chi_square) has a p-value less than 0.05, implying the null hypothesis is rejected. Accordingly, the study finds a unidirectional causality running from tourism development, and human development to income inequality in Singapore. Overall, these findings are in line with the outcome of regression analysis. Hence, it allows us to conclude that our findings are convincing to suggest policies.

Table 7: Results of the causality test

Null hypothesis: No causality	Chi_square	p-value
lnTO does not Granger cause *IE*	8.421	0.0345
IE does not Granger cause *lnTO*	2.471	0.7809
lnGDP does not Granger cause *IE*	1.329	0.9319
IE does not Granger cause *lnGDP*	4.657	0.4591
Glob does not Granger cause *IE*	4.710	0.3183
IE does not Granger cause *Glob*	6.357	0.1740
HC does not Granger cause *IE*	7.296	0.0451
IE does not Granger cause *HC*	2.460	0.6517

Discussion

The results of our study provide strong evidence that international visitors play a crucial role in reducing income inequalities in Singapore. This conclusion aligns with previous research conducted by Njoya and Seetaram [11] in Kenya, Li, Chenet al. [53] in China, and Uzar and Eyuboglu [14] in Turkey. However, it is important to note that our findings are inconsistent with the research conducted by Alam and Paramati [54]. One of the key contributions of our study is showcasing that the impact of tourism on income inequality is asymmetric, both in the short- and long-term. To understand these findings, some reasons are given:

Firstly, Singapore boasts an effective government and strong institutional frameworks. This dynamic governance creates ample opportunities for economic activities, including tourism and financial services. Furthermore, the stability provided by these institutions attracts a significant number of visitors to the country [5, 55].

Secondly, Singapore's robust tax laws ensure that tax evasion and avoidance are not prevalent. This strict approach contributes to a fair economic environment, which ultimately benefits income distribution [56].

Lastly, Singapore's well-developed education system plays a vital role [23]. It offers individuals a wide range of employment opportunities with higher incomes. However, it is worth noting that a considerable portion of the labor force in the tourism industry in Singapore is self-employed or engaged in family businesses. As a result, the benefits of tourism primarily accrue to the owners, which may explain why Singapore's income inequality index is not ranked among the lowest in the world. In conclusion, our research sheds light on the substantial impact of international visitors on reducing income inequalities in Singapore. The country's effective government, strong tax laws, and developed education system contribute to this positive dynamic. These findings have significant implications for policymakers and stakeholders interested in promoting a more equitable society in Singapore and beyond.

5. Conclusion and policy implications

This study is conducted to probe the symmetric and asymmetric effect of tourism development on income inequality in Singapore, including economic growth, globalization, and human development in the models. To achieve this goal, first, the study employed the unit root tests proposed by Dickey and Fuller [57], Phillips and Perron [58], and Lee and Strazicich [51] with two structural breaks. Next, the bound test technique introduced by Pesaran, Shin and Smith [52] was applied to assess the symmetric and asymmetric cointegration. Afterward, the symmetric ARDL method proposed by Pesaran and Shin [49], and the asymmetric ARDL approach given by Shin, Yu and Greenwood-Nimmo [50] were utilized to estimate the short- and long-run coefficients. Finally, the Wald test was adopted to conclude that the impact of tourism development on income inequality in Singapore is symmetry or asymmetry.

With a strict econometric strategy, our outcome provides some useful findings. *Firstly*, the study concludes that the impact of tourism on income inequality is asymmetric, and positive changes are greater than negative changes. More precisely, the impact of an increase in tourism development on income inequality has a larger effect than a decrease in tourism development. Moreover, the causal test confirms a unidirectional causality from tourism development to income inequality. *Secondly*, the study also indicates that increasing economic growth and globalization might increase income inequality. *Thirdly*, improving human capital stimulates income distribution more equally in Singapore. Furthermore, there is a unidirectional causality running from human capital to income inequality.

Policy implications

Based on the findings, the study delineates several significant policies that would considerably be helpful for Singapore’s government to reduce income inequality by developing the tourism industry, improving human capital, and maintaining sustainable development.

First and foremost, we suggest that policy-makers in Singapore should maintain appropriate policies to strengthen the tourism industry and encourage community-based tourism initiatives. That means government should empower local communities to participate in decision-making, providing training and capacity-building programs, and promoting the development of small-scale enterprises and cooperative ventures. Besides, replacing outdated regulations and laws is necessary, especially in tax and transfer policies in tourism. Some policies, such as minimum wage regulations, collective bargaining rights, and workplace safety standards, also help reduce income inequality in Singapore. In addition, we suggest that Singapore's administrators focus on providing vocational training, promoting entrepreneurship, and fostering partnerships between tourism industry stakeholders and educational institutions. By equipping individuals with the necessary skills and knowledge, they can access higher-paying jobs within the tourism sector, reducing income disparities.

Finally, enhancing economic growth and expanding globalization must be accompanied by the improvement of human capital in order to mitigate the potential increase in income inequality. The government of Singapore should consider implementing social safety nets and welfare programs to reduce income inequality and provide support for vulnerable segments of society. These initiatives are essential to provide a safety net for individuals and families facing economic hardships, through targeted cash transfers, unemployment benefits, healthcare assistance, and other social services. By doing so, Singapore's government can ensure a basic level of social protection for its citizens and mitigate the adverse effects of income inequality. These actions will lead to a more inclusive society and promote more equitable economic growth. By supporting those in need and reducing income disparities, the government can contribute to a fairer distribution of resources and opportunities. It is important to note that the design and implementation of these programs should be tailored to the needs and context of Singapore, taking into account factors such as affordability, sustainability, and the overall impact on the economy.

Even though significant empirical evidence is acknowledged in this study, we agree that it still has some limits. In general, many macroeconomic variables can affect income distribution in Singapore. Thus, some related economic variables, such as foreign direct investment, institutional quality, and the development of the national education system, should be further considered. Additionally, future research might employ our model in other regions to help policy-makers systematically understand the role of tourism development, economic growth, and human capital in income distribution and strengthen the belief of administrators in tourism development. We also suggest that several novel econometric techniques, such as the time-varying parameter

VAR model, and quantile on quantile approach, should be applied to support policy-makers with a comprehensive policy guideline./.

Reference

1. Alvaredo F, Chancel L, Piketty T, Saez E, Zucman G. World Inequality Report 2018. 2018.

2. Hu Z. "The effect of income inequality on human capital inequality: Evidence from China", *Structural Change and Economic Dynamics*, vol. 58, no. pp. 471-89, 2021.

3. Nguyen CP, Schinckus C, Su TD, Chong FHL. "The Influence of Tourism on Income Inequality", *Journal of Travel Research*, vol. 60, no. 7, pp. 1426-44, 2020.

4. Subramaniam Y, Masron TA, Loganathan N. "Tourism and income inequality", *Journal of Business and Socio-economic Development*, vol. 2, no. 2, pp. 181-94, 2022.

5. Ngoc BH, Hai LM. "Time-frequency nexus between tourism development, economic growth, human capital, and income inequality in Singapore", *Applied Economics Letters*, vol. 31, no. 4, pp. 259-64, 2024.

6. Fang J, Gozgor G, Paramati SR, Wu W. "The impact of tourism growth on income inequality: Evidence from developing and developed economies", *Tourism Economics*, vol. 27, no. 8, pp. 1669-91, 2020.

7. Lee C-C, Chen M-P, Xing W. "Do national cultures matter for tourism development? Some international evidence", *Economic Analysis and Policy*, vol. 74, no. pp. 666-86, 2022.

8. Ohlan R. "The relationship between tourism, financial development and economic growth in India", *Future Business Journal*, vol. 3, no. 1, pp. 9-22, 2017.

9. Qiao G, Chen Q. "Tourism and inequality: problems and prospects", *Journal of Tourism and Cultural Change*, vol. 19, no. 5, pp. 732-8, 2021.

10. Carrascal Incera A, Fernández MF. "Tourism and income distribution: Evidence from a developed regional economy", *Tourism Management*, vol. 48, no. pp. 11-20, 2015.

11. Njoya ET, Seetaram N. "Tourism Contribution to Poverty Alleviation in Kenya: A Dynamic Computable General Equilibrium Analysis", *J Travel Res*, vol. 57, no. 4, pp. 513-24, 2018.

12. Kinyondo A, Pelizzo R. "Tourism, development and inequality: The case of Tanzania", *Poverty & Public Policy*, vol. 7, no. 1, pp. 64-79, 2015.

13. Khan A, Bibi S, Lyu J, Raza A, Hayat H, Meo MS. "Unraveling the Nexuses of Tourism, Terrorism, and Well-Being: Evidence from Pakistan", *Journal of Hospitality & Tourism Research*, vol. 44, no. 6, pp. 974-1001, 2020.

14. Uzar U, Eyuboglu K. "Can tourism be a key sector in reducing income inequality? An empirical investigation for Turkey", *Asia Pacific Journal of Tourism Research*, vol. 24, no. 8, pp. 822-38, 2019.

15. Porto N, Espinola N. "Labor income inequalities and tourism development in Argentina: A regional approach", *Tourism Economics*, vol. 25, no. 8, pp. 1265-85, 2019.

16. Shahbaz M, Solarin SA, Azam M, Tiwari AK. "Tourism-induced income distribution in Malaysia: a practical experience of a truly Asian economy", *Current Issues in Tourism*, vol. 23, no. 23, pp. 2910-29, 2019.

17. World Travel & Tourism Council. Economic Impact Reports - Singapore 2020 [cited April 4, 2021]. Available from: https://wttc.org/Research/Economic-Impact/moduleId/704/itemId/200/controller/DownloadRequest/action/QuickDownload.

18. Oviedo-García MÁ, González-Rodríguez MR, Vega-Vázquez M. "Does Sun-and-Sea All-Inclusive Tourism Contribute to Poverty Alleviation and/or Income Inequality Reduction? The Case of the Dominican Republic", *Journal of Travel Research*, vol. 58, no. 6, pp. 995-1013, 2018.

19. Yan Y, Shah MI, Sharma GD, Chopra R, Fareed Z, Shahzad U. "Can tourism sustain itself through the pandemic: nexus between tourism, COVID-19 cases and air quality spread in the 'Pineapple State' Hawaii", *Current Issues in Tourism*, vol. 25, no. 3, pp. 421-40, 2021.

20. Karabulut G, Bilgin MH, Demir E, Doker AC. "How pandemics affect tourism: International evidence", *Ann Tour Res*, vol. 84, no. pp. 102991, 2020.

21. Yang Y, Zhang H, Chen X. "Coronavirus pandemic and tourism: Dynamic stochastic general equilibrium modeling of infectious disease outbreak", *Ann Tour Res*, vol. 83, no. pp. 102913, 2020.

22. Işik C, Kasımatı E, Ongan S. "Analyzing the causalities between economic growth, financial development, international trade, tourism expenditure and/on the CO2emissions in Greece", *Energy Sources, Part B: Economics, Planning, and Policy*, vol. 12, no. 7, pp. 665-73, 2017.

23. Khoi NH, Le NH, Ngoc BH. "The effect of tourism development on the ecological footprint in Singapore: evidence from asymmetric ARDL method", *Current Issues in Tourism*, vol. 25, no. 15, pp. 2500-17, 2021.

24. Katrakilidis C, Trachanas E. "What drives housing price dynamics in Greece: New evidence from asymmetric ARDL cointegration", *Economic Modelling*, vol. 29, no. 4, pp. 1064-9, 2012.

25. Ahmed Z, Zhang B, Cary M. "Linking economic globalization, economic growth, financial development, and ecological footprint: Evidence from symmetric and asymmetric ARDL", *Ecological Indicators*, vol. 121, no. pp. 107060, 2021.

26. Kuznets S. "Economic Growth and Income Inequality", *The American Economic Review*, vol. 45, no. 1, pp. 1-28, 1955.

27. Braun M, Parro F, Valenzuela P. "Does Finance Alter the Relation between Inequality and Growth?", *Economic Inquiry*, vol. 57, no. 1, pp. 410-28, 2019.

28. Royuela V, Veneri P, Ramos R. "The short-run relationship between inequality and growth: evidence from OECD regions during the Great Recession", *Regional Studies*, vol. 53, no. 4, pp. 574-86, 2018.

29. Breunig R, Majeed O. "Inequality, poverty and economic growth", *International Economics*, vol. 161, no. pp. 83-99, 2020.

30. Scholl N, Klasen S. "Re-estimating the relationship between inequality and growth", *Oxford Economic Papers*, vol. 71, no. 4, pp. 824-47, 2019.

31. Brueckner M, Lederman D. "Inequality and economic growth: the role of initial income", *Journal of Economic Growth*, vol. 23, no. 3, pp. 341-66, 2018.

32. Benos N, Karagiannis S. "Inequality and Growth in the United States: Why Physical and Human Capital Matter", *Economic Inquiry*, vol. 56, no. 1, pp. 572-619, 2018.

33. Chi J. "Revisiting the tourism-inequality nexus: evidence from a panel of developed and developing economies", *Current Issues in Tourism*, vol. 24, no. 6, pp. 755-67, 2020.

34. Dwyer L. "Globalization of tourism: Drivers and outcomes", *Tourism Recreation Research*, vol. 40, no. 3, pp. 326-39, 2015.

35. Ghosh S, Mitra SK. "Tourism and inequality: A relook on the Kuznets curve", *Tourism Management*, vol. 83, no. pp. 2021.

36. Ulucak R, Danish, Li N. "The nexus between economic globalization and human development in Asian countries: an empirical investigation", *Environmental Science and Pollution Research*, vol. 27, no. 3, pp. 2622-9, 2020.

37. Saud S, Chen S, Haseeb A, Sumayya. "The role of financial development and globalization in the environment: Accounting ecological footprint indicators for selected one-belt-one-road initiative countries", *Journal of Cleaner Production*, vol. 250, no. pp. 2020.

38. Tsaurai K. "Influence of Tourism on Income Inequality in Transitional Economies: Does Foreign Direct Investment Matter?", *EuroEconomica*, vol. 41, no. 1, pp. 76-89, 2022.

39. Becker GS. "Investment in Human Capital: A Theoretical Analysis", *Journal of Political Economy*, vol. 70, no. 5, pp. 9-49, 1962.

40. Ajide KB, Alimi OY. "Income inequality, human capital and terrorism in Africa: Beyond exploratory analytics", *International Economics*, vol. 165, no. pp. 218-40, 2021.

41. Chani MI, Jan SA, Pervaiz Z, Chaudhary AR. "Human capital inequality and income inequality: testing for causality", *Quality & Quantity*, vol. 48, no. 1, pp. 149-56, 2012.

42. Suhendra I, Istikomah N, Ginanjar RAF, Anwar CJ. "Human Capital, Income Inequality and Economic Variables: A Panel Data Estimation from a Region in Indonesia", *The Journal of Asian Finance, Economics and Business*, vol. 7, no. 10, pp. 571-9, 2020.

43. Ofori IK, Dossou TAM, Akadiri SS. "Towards the quest to reduce income inequality in Africa: is there a synergy between tourism development and governance?", *Current Issues in Tourism*, vol., no. pp. 1-21, 2022.

44. Lv Z. "Deepening or lessening? The effects of tourism on regional inequality", *Tourism Management*, vol. 72, no. pp. 23-6, 2019.

45. Lee J-W, Lee H. "Human capital and income inequality*", *Journal of the Asia Pacific Economy*, vol. 23, no. 4, pp. 554-83, 2018.

46. Chishti MZ, Ullah S, Ozturk I, Usman A. "Examining the asymmetric effects of globalization and tourism on pollution emissions in South Asia", *Environmental Science and Pollution Research*, vol. 27, no. 22, pp. 27721-37, 2020.

47. Mahadevan R, Suardi S, Ji C, Hanyu Z. "Is urbanization the link in the tourism–poverty nexus? Case study of China", *Current Issues in Tourism*, vol., no. pp. 1-15, 2021.

48. Croes R, Ridderstaat J, Bąk M, Zientara P. "Tourism specialization, economic growth, human development and transition economies: The case of Poland", *Tourism Management*, vol. 82, no. pp. 2021.

49. Pesaran MH, Shin Y. An Autoregressive Distributed Lag Modelling Approach to Cointegration Analysis: Department of Applied Economics, University of Cambridge; 1995.

50. Shin Y, Yu B, Greenwood-Nimmo M. Modelling Asymmetric Cointegration and Dynamic Multipliers in a Nonlinear ARDL Framework. In: Sickles RC, Horrace WC, editors.

Festschrift in Honor of Peter Schmidt, Econometric Methods and Applications. New York, NY: Springer; 2014. p. 281-314.

51. Lee J, Strazicich MC. "Minimum Lagrange Multiplier Unit Root Test with Two Structural Breaks", *Review of Economics and Statistics*, vol. 85, no. 4, pp. 1082-9, 2003.

52. Pesaran MH, Shin Y, Smith RJ. "Bounds testing approaches to the analysis of level relationships", *Journal of Applied Econometrics*, vol. 16, no. 3, pp. 289-326, 2001.

53. Li H, Chen JL, Li G, Goh C. "Tourism and regional income inequality: Evidence from China", *Annals of Tourism Research*, vol. 58, no. pp. 81-99, 2016.

54. Alam MS, Paramati SR. "The impact of tourism on income inequality in developing economies: Does Kuznets curve hypothesis exist?", *Annals of Tourism Research*, vol. 61, no. pp. 111-26, 2016.

55. Azam M, Mahmudul Alam M, Haroon Hafeez M. "Effect of tourism on environmental pollution: Further evidence from Malaysia, Singapore and Thailand", *Journal of Cleaner Production*, vol. 190, no. pp. 330-8, 2018.

56. Agiomirgianakis G, Serenis D, Tsounis N. "Effective timing of tourism policy: The case of Singapore", *Economic Modelling*, vol. 60, no. pp. 29-38, 2017.

57. Dickey DA, Fuller WA. "Likelihood ratio statistics for autoregressive time series with a unit root", *Econometrica*, vol. 49, no. 4, pp. 1057-72, 1981.

58. Phillips PCB, Perron P. "Testing for a unit root in time series regression", *Biomètrika*, vol. 75, no. 2, pp. 335-46, 1988.
