# Supplementary material for: Do positive and negative shocks of tourism development affect income inequality in a developed country?
Source: PLoS One. 2024 Apr 16;19(4):e0301628. doi: 10.1371/journal.pone.0301628 (PMC11020903; doi:10.1371/journal.pone.0301628)
Supplement: Attachment — Submitted filename: Response to Editor-in-Chief & Reviewers.docx [file pone.0301628.s002.docx]

**Overall Modifications**

The authors thank Prof. Abdullah Mohammad Ghazi Al khatib - the Academic Editor of Plos One journal, and the two esteemed reviewers for their valuable comments. We regret some weakness of the original manuscript. In this revision, The authors have tried to address every comment and suggestion. All modified parts are indicated in **green** in the new version of the manuscript. The authors sincerely hope that the revised manuscript meets the expectations of the Editors and Reviewers.

Q1. Clearly lay out the specific research questions you are investigating based on the relationships you are exploring.

A1. The experience and sharing of reviewers is highly appreciated. We have tried to improve the introduction section. We hope that it makes the problem research clear. Please see page 4 in the revised manuscript or the below text:

“Singapore presents itself as a captivating context for investigating this association. Analysis of the relationship between tourism development and income inequality is essential for Singapore for the following reasons. First, the tourism industry generated around 527,500 jobs and contributed SGD 52.5 billion to the economy in 2020 [17]. Singapore's tourism development is an enticing example for emerging countries, as it enhances per capita income. Singapore, currently ranked among the top 10 countries in the world with the highest income, boasts an impressive per capita income of $60,729 per year (at the fixed price of 2010). However, it is important to note that such prosperity does not necessarily address the prevailing income disparity between the affluent and the less fortunate within Singapore. Startling figures from a recent report on income inequality indicate that in 2010, the income gap between the higher and lower-income classes was $9,288. Shockingly, this gap has widened to $12,840 in 2020, highlighting a growing challenge of eradicating inequality for Singaporean administrators. Second, the significance of tourism section has long been identified, so the Singaporean government has consistently devoted resources to fostering the growth of this industry. At the same time, Singapore has a diverse and well-integrated culture. These characteristics explain Singapore's spectacular achievement in tourism development, nowadays, it is a top-visited market in the world. Therefore, an analysis of the impact of tourism development on income distribution in Singapore might provide a practical lesson for emerging countries in planning tourism development strategies. Due to the two above reasons, the study is conducted to answer the research questions below:

Does tourism development affect income distribution in Singapore?

Is it the symmetric or asymmetric impact? If the asymmetric influence is found, how do positive and negative changes in tourism development affect income inequality?.”

Q2. Identify a gap in the existing literature that your study aims to fill, and explain how your work addresses that gap.

A2. Thanks for this comment. The authors have edited the revised manuscript to better highlight a gap in the existing literature and the specific contribution of this research. For more details, please see page 10 or below text:

“. Katrakilidis and Trachanas [43], and Ahmed, Zhang and Cary [44] pointed out that if the relationship between these variables is asymmetric, the policy implications based on the linear framework could be unreliable. Hence, this work aims to explore the role of tourism in reducing IE in Singapore by considering economic growth, globalization, and human capital in which tourism activities occur. The contribution of this study are summarized in detail:

(i) Despite the abundance of previous studies on links between tourism and income inequality, the negative and positive changes in tourism development that might lead to effects of different magnitude or directions in income have been ignored. If the nexus between TO and IE is asymmetric, the policy implications based on symmetric methods can be unreliable. This study employs the linear ARDL and non-linear ARDL approaches to identify a symmetric or asymmetric effect of tourism on income inequality to provide a comprehensive understanding of tourism development in Singapore.
(ii) Globalization and human capital are considered the keys to tourism development. However, the impact of two factors on income inequality is still fragmented and unclear in Singapore. In this study, globalization and human capital play as control variables in identifying the tourism-income inequality nexus.
(iii) From 1978 to 2019, Singapore’s economy suffered two financial crises (in 1997 and 2008), implying that the data might have some structural breaks. Therefore, an advanced unit root technique was also employed to probe structural breaks in the data, which is superior to many other tests regarding size and accuracy in identifying breaks. Along with the results obtained from the non-linear ARDL approach, the findings of this study strengthen administrators’ belief in promoting tourism and reducing income inequality in Singapore.

Q3. Formulate hypotheses for each independent variable, grounded in theoretical literature. Shorten and tighten up the Introduction and Literature Review sections as suggested.

A3. We thank your observation. In this version, four hypotheses are postulated. The Introduction and Literature review sections were also modified to shorten and tighten. Please see the revised manuscript for more details.

Q4. Justify why you analyzed data from the 1978-2019 period specifically.

A4. Thanks for this comment. We updated the data. The new length of data is from 1978 to 2022. This action leads to changes in result tests and estimated coefficients. However, the findings of this study do not change.

Q5. Include and describe a correlation matrix in this section or the Results.

A5. Yes, we added the correlation matrix in Table 3 in the revised manuscript.

Q6. In the Discussion, compare and contrast your long-term findings in detail with the empirical results from other relevant prior studies.

A6. We fully agree with this comment. The discussion section has been modified to compare our findings to those of relevant prior studies. It makes our contribution clear. Please see pages 19-20 for more details, or below text:

“The empirical results suggest that international visitors contribute to reducing income inequalities in Singapore. That means hypothesis 2 is accepted. This finding is in line with Njoya and Seetaram [11] for Kenya, Li, Chenet al. [56] for China, and Uzar and Eyuboglu [14] for Turkey. However, it is inconsistent with Alam and Paramati [57]. The main contribution of this study shows that the influence of tourism on income inequality is asymmetric both in the short- and long-run. To explain for these findings, some reasons are given: (*i*) Singapore has an effective government and better institutions. It is easy to recognize that a dynamic government might create more opportunities for economic activities, such as tourism and financial services, while institutional security might attract more visitors [3]; (*ii*) Singapore has strong tax laws. Therefore, the tax evasion and tax avoidance acts are not fully valid in Singapore [58]; (*iii*) Singapore has a developed education system, giving individuals more jobs and a better income. However, a significant part of the labor force in the tourism industry in Singapore is involved in self-employment or family business, and tourism only benefits the owners. Hence, this might explain why the income inequality index of Singapore is not ranked the lowest in the world.

The positive coefficient of the *lnGDP* variable suggests that economic growth enhances income inequality in Singapore, implying that hypothesis 1 is also accepted. The finding is also similar to several studies, such as Scholl and Klasen [22], Benos and Karagiannis [24], and Elveren and Özgür [59]. In fact, Singapore has implemented several policies and initiatives to address income inequality and promote economic growth. For example, Singapore’s government has reformed tax policies and applied progressive taxation. This tax system follows a progressive structure, with higher-income earners paying a higher percentage of taxes. Another policy is the “SkillsFuture” program issued by the government, which provides training subsidies and support for individuals to upgrade their skills and stay relevant in the evolving job market. However, the increase in economic growth in the past few decades changed the income structure of individuals and classes. Despite the government’s efforts, income inequality remains an ongoing challenge, and further measures may be needed to create a more equitable distribution of income and wealth in Singapore.

The finding also indicates that hypothesis 4 is accepted, and globalization positively drives income inequality in Singapore. This conclusion aligns with Haseeb, Suryantoet al. [60], Chishti, Ullahet al. [61]. Cristiano and İpek [62] noted that governance institution is a necessary condition to reduce the negative impact of globalization on inequality. In fact, Singapore's economy heavily depends on trade and foreign investment. So, Singapore’s administrators have implemented policies and initiatives to create an attractive business environment, including favorable tax regimes, ease of doing business, and robust legal and regulatory frameworks to attract multinational corporations. Besides, Singapore’s government has actively pursued free trade agreements with various countries and regions worldwide, emphasizing connectivity and innovation to enhance market access and reduce trade barriers, allowing Singaporean businesses to tap into global markets. However, globalization can widen income disparities within and between individuals [60] due to those with better access to education, technology, and capital are more likely to benefit from globalization, while those with limited access to such resources may be left behind.

Hypothesis 3 is also confirmed, and human development stimulates income equality in Singapore. This is not surprising since Singapore’s human capital per person index has rapidly increased along with GDP per capita. In the long term, Singapore has achieved high levels of income per capita, low unemployment rates, and a high standard of living. This country also has invested heavily in education, ensuring access to quality schooling for all citizens. Nowadays, Singapore has a well-developed public education system, which ensures holistic human development for all Singaporeans. Moreover, scholarships, bursaries, and financial aid programs are available to support students from lower-income backgrounds. This policy helps to reduce unequal access and improve income for individuals and workers”.

Q7. Provide a thorough discussion of the study's limitations. Offer recommendations for future research building on your work.

A7. The study’s limitation section has also been revised. We revealed several limitations of this study and suggested some directions for future research. Please see the lowest paragraph of the revised manuscript or the below text:

“Even though significant empirical evidence is acknowledged in this study, we agree that it still has some limits. In general, many macroeconomic variables can affect income distribution in Singapore. Thus, some related economic variables, such as foreign direct investment, institutional quality, and the development of the national education system, should be further considered. Furthermore, this research does not delve into the structure of variables (lead-lag variable) and heteroskedasticity impacts of tourism development through quantile analysis. Therefore, future studies should utilize novel econometric techniques such as the time-varying parameter vector autoregressive model and the quantile-on-quantile approach to provide a more comprehensive understanding of the role of tourism development, economic growth, and human capital in income distribution across various contexts. These recommendations are essential for supporting policy-makers in making informed decisions”.

Q8. Consider incorporating the 3 additional references suggested by Reviewer 1.

A8. Yes, four new articles were added, including three suggested references of Reviewer 1.

Q9. Highlight the key findings more prominently in the Abstract section.

A9. Yes, we agree with this comment. The Abstract section was modified. Please see the first page of the revised manuscript.

Finally, some grammatical and structural errors were found and edited. The authors sincerely hope that the revised manuscript meets the expectations of the Academic Editor and Reviewer.
